# Serum neutralization activity declines but memory B cells persist after cure of chronic hepatitis C

**DOI:** 10.1038/s41467-022-33035-z

**Published:** 2022-09-16

**Authors:** Akira Nishio, Sharika Hasan, Heiyoung Park, Nana Park, Jordan H. Salas, Eduardo Salinas, Lela Kardava, Paul Juneau, Nicole Frumento, Guido Massaccesi, Susan Moir, Justin R. Bailey, Arash Grakoui, Marc G. Ghany, Barbara Rehermann

**Affiliations:** 1 Immunology Section, Liver Diseases Branch, National Institute of Diabetes and Digestive and Kidney Diseases, National Institutes of Health, DHHS, Bethesda, MD 20892 USA; 2 Department of Medicine, Johns Hopkins University School of Medicine, Baltimore, MD 21205 USA; 3 Division of Infectious Diseases, Emory Vaccine Center, Division of Microbiology and Immunology, Emory University School of Medicine, Atlanta, GA 30322 USA; 4 Emory National Primate Research Center, Emory Vaccine Center, Atlanta, GA 30329 USA; 5 Laboratory of Immunoregulation, National Institute of Allergy and Infectious Diseases, National Institutes of Health, DHHS, Bethesda, MD 20892 USA; 6 Division of Data Services, NIH Library, Office of Research Services, National Institutes of Health, Bethesda, MD USA; 7 Contractor- Zimmerman Associates, Inc, Fairfax, VA USA; 8 Clinical Research Section, Liver Diseases Branch, National Institute of Diabetes and Digestive and Kidney Diseases, National Institutes of Health, DHHS, Bethesda, MD 20892 USA

**Keywords:** Translational research, Antibodies, Hepatitis C virus, Immunological memory

## Abstract

The increasing incidence of hepatitis C virus (HCV) infections underscores the need for an effective vaccine. Successful vaccines to other viruses generally depend on a long-lasting humoral response. However, data on the half-life of HCV-specific responses are lacking. Here we study archived sera and mononuclear cells that were prospectively collected up to 18 years after cure of chronic HCV infection to determine the role of HCV antigen in maintaining neutralizing antibody and B cell responses. We show that HCV-neutralizing activity decreases rapidly in potency and breadth after curative treatment. In contrast, HCV-specific memory B cells persist, and display a restored resting phenotype, normalized chemokine receptor expression and preserved ability to differentiate into antibody-secreting cells. The short half-life of HCV-neutralizing activity is consistent with a lack of long-lived plasma cells. The persistence of HCV-specific memory B cells and the reduced inflammation after cure provide an opportunity for vaccination to induce protective immunity against re-infection.

## Abstract (web summary)

Long-term dynamics of the humoral response to HCV in cured individuals aren’t well understood. Here, Nishio et al. show that virus-neutralizing antibody levels decrease in potency and breadth after cure of chronic hepatitis C, while HCV-specific memory B cells persist.

## Introduction

Hepatitis C virus (HCV) infection remains a global health issue. Approximately 70 million people are chronically infected worldwide, and at least 400,000 people die annually because of HCV-related diseases, namely liver cirrhosis, liver failure, and hepatocellular carcinoma^1^. Despite the recent development of highly efficient direct-acting antivirals (DAA) that cure HCV infection^2^, most countries lag behind the World Health Organization mandate to eliminate the public health threat of HCV infection by 2030^3^. While HCV elimination requires a reduction of new HCV infections by 90%, the US experienced an increase in the rate of new infections in recent years, and more than 1.75 million new infections occur per year worldwide^4^. Therefore, global control of HCV requires a vaccine to prevent both de novo infections of naïve individuals and re-infections of those who cleared HCV with antiviral therapy^5–7^.

Previous work defined successful immune responses to HCV in patients and non-human primates that spontaneously cleared the acute infection. Collectively, these studies demonstrated that viral clearance is associated with HCV-specific T cells and neutralizing antibodies^8–13^. Studies in non-human primates have further shown that recovery from acute HCV infection can result in protection upon re-exposure to HCV^14–17^. Protection was associated with strong T cell responses. In contrast, those who develop chronic infection have weak and narrow HCV-specific T cell responses that rapidly exhaust and become functionally impaired^18–20^.

The first candidate vaccine against HCV was recently evaluated in a large, multicenter phase 1/2 study of people at risk for HCV infection^21^. This vaccine consisted of a prime/boost regimen with vectors that encode the HCV nonstructural proteins for induction of antiviral T cells and lacked targets for neutralizing antibodies. While the vaccine was safe and induced HCV-specific T cell responses, it did not prevent chronic infection^21^. This result has shifted the focus on humoral immune responses for HCV vaccine development with the goal to induce broadly cross-neutralizing responses^22,23^. Indeed, nearly all licensed vaccines in use today confer protective immunity through the induction of neutralizing antibodies.

Humoral immune responses of subjects who spontaneously clear acute HCV infection and subsequent re-infections do not target unique epitopes^24,25^, but appear earlier and are broader than those in subjects that develop chronic infection^12,26^. Accordingly, passive transfer of neutralizing antibodies has been shown to protect from HCV infection^27–29^. Broadly cross-neutralizing antibodies target five antigenic regions (AR). AR1, AR2, and AR3 are located on the HCV envelope 2 (E2) protein, and AR4 and AR5 contain epitopes that have amino acids on both E1 and E2 proteins^30,31^. Such responses are also found in patients with established chronic HCV infection^26,32,33^, but they do not clear the autologous HCV strain, which has escaped via the selection of quasispecies with mutations that are not recognized^34^.

Successful humoral immune responses require the induction of memory B cells and long-lived plasma cells^35^. The first exposure to virus or antigen induces the interaction between B cells and follicular T cells, which results in the differentiation of B cells into memory B cells and long-lived plasma cells. Long-lived plasma cells reside in the bone marrow and are thought to be the main source of long-lasting antibody responses in the blood^36–38^. Indeed, natural and vaccine-induced antibody responses to many common viruses such as varicella-zoster virus, measles, and mumps viruses are known to have half-lives of greater than 50 and 200 years, respectively^38^. Memory B cells produce antibodies with less avidity but a broader range^35,39–42^. They are enriched for cells with an atypical phenotype in chronic viral infection^43,44^ and are just very recently being explored as an alternative target for vaccination^45^. The selection of antigen-specific B cells into the memory B cell fate with the exit of the germinal center as opposed to plasmablast/plasma cell fate is still not well understood^46^. At present, the longevity of the HCV-specific antibody response and the relative contribution of plasma cells versus memory cells in maintaining such responses are unknown.

In this work, we define the longevity, potency, and breadth of humoral immune responses against HCV in patients that have been followed in our institute with serially collected serum and lymphocyte samples for up to 18 years after treatment-induced viral clearance, i.e., almost since the approval of interferon-alpha as the first treatment for HCV in 1991 [19, 20]. We show that patients with chronic hepatitis C display potent humoral responses that cross-neutralize four HCV strains and nineteen HCV pseudoparticles with different E1E2 sequences. After treatment-induced HCV clearance, serum neutralizing activity declines rapidly (half-life: 4.8–7.3 years), and neutralization of more than half of the HCVpp is lost within 6–10 years. Despite the short-lived humoral response, HCV-specific memory B cells persist for decades after HCV clearance and exhibit a restored resting phenotype, normalized chemokine receptor expression, and preserved ability to differentiate into antibody-secreting cells. In conclusion, the serum neutralizing activity against HCV is much shorter than that against other viruses. This suggests a lack of long-lived HCV-specific plasma cells in the bone marrow. The persistence of HCV-specific memory B cells and the reduced inflammation after viral clearance provide an opportunity to induce protective immunity by vaccination to prevent re-infection. The availability of effective antiviral therapy and the long-term follow-up of treated patients also make HCV infection a useful model to understand and restore functional humoral immune responses.

## Results

### HCV-neutralizing activity declines after cure of chronic hepatitis C

**Fig. 1:**
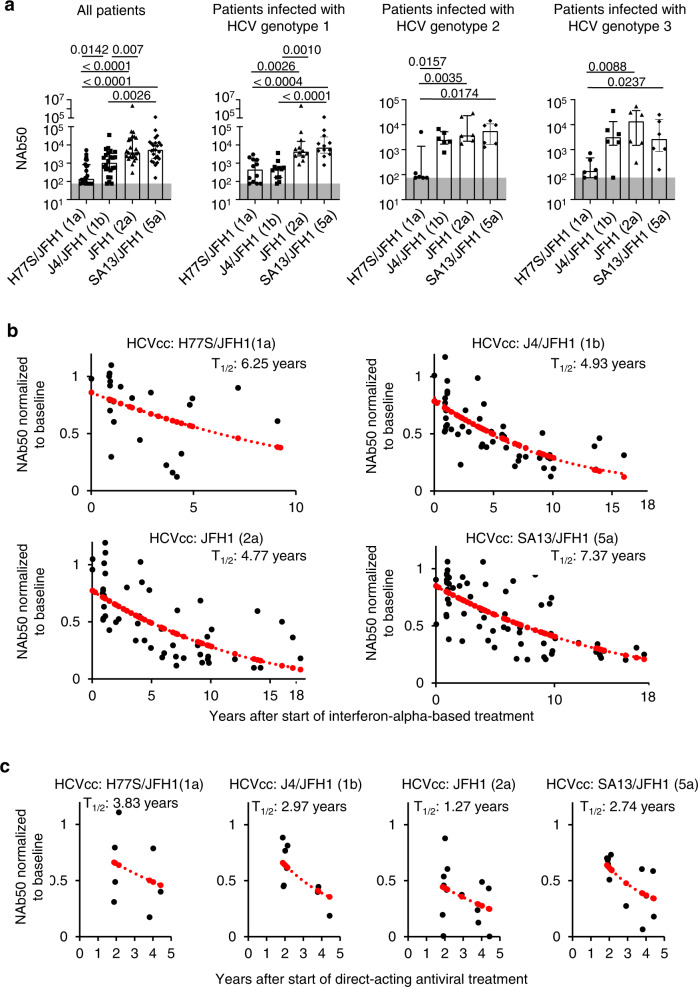
HCV-neutralizing activity declines after cure of chronic hepatitis C. Neutralizing activity of sera from patients with chronic HCV genotype 1, 2, or 3 infections. Neutralization was tested against cell culture-produced hepatitis C viruses (HCVcc) with JFH1 backbone and core-NS2 sequences of H77S (genotype 1a), J4 (genotype 1b), JFH1 (genotype 2a), and SA13 (genotype 5a) sequence. **a** NAb50 (50% neutralizing activity) is shown for sera from all patients (*n* = 24), or stratified by chronic HCV genotype 1 (*n* = 12), genotype 2 (*n* = 6) or genotype 3 infection (*n* = 6). Each symbol represents a patient; error bars show median + IQR. Two-sided *p* values are shown and were calculated using a linear mixed model based on the log-transformation of the outcome measurement with Tukey–Kramer procedure as a post hoc test was performed. The upper line of the shaded area represents the threshold of quantitation (NAb50 = 75). **b**, **c** NAb50 of serial serum samples of patients. Neutralization was tested against HCVcc with H77S/JFH1, J4/JFH1, JFH1, and SA13/JFH1 sequences and normalized to the baseline (chronic HCV infection prior to treatment, *t* = 0) value. The half-life (T_1/2_) of NAb50 was calculated using a simple linear model of exponential decay, with the dependent variable normalized for each patient’s baseline value. Normalized NAb50 values that were predicted by mathematical modeling (see methods section) are shown as red dots. Source data are provided as a Source Data file. **b** Each data point represents the NAb50 of a serum sample obtained at the indicated time after interferon-alpha-based cure of chronic HCV infection. Twenty-seven (*n* = 27) serum samples were tested for neutralization of H77S/JFH1 HCVcc; *n* = 60 serum samples tested for neutralization of J4/JFH1 HCVcc; *n* = 68 serum samples tested for neutralization of JFH1 HCVcc and *n* = 71 serum samples tested for neutralization of SA13/JFH1 HCVcc. **c** Each data point represents the NAb50 of a serum sample obtained at the indicated time after direct-acting antiviral (DAA) therapy with *n* = 7 serum samples tested for neutralization of H77S/JFH1 HCVcc; *n* = 9 serum samples tested for neutralization of J4/JFH1 HCVcc; *n* = 13 serum samples tested for neutralization of JFH1 HCVcc and *n* = 13 serum samples tested for neutralization of SA13/JFH1 HCVcc.

We started this study by assessing serum samples from 24 patients with chronic HCV infection (Table 1) for their ability to neutralize a panel of four cell culture-produced recombinant hepatitis C viruses (HCVcc). These four HCVcc share the HCV-JFH1 strain backbone and differ in the envelope proteins that they encode. The envelope proteins are derived from either HCV genotype 1a (HCV H77 strain), 1b (HCV J4 strain), 2a (HCV-JFH1 strain), or 5a (HCV SA13 strain). Irrespective of the HCV genotype the patients were infected with, each patient’s serum neutralized multiple HCVcc with a 50% neutralizing activity (NAb50) between 75 and 2,211,000 (median: 2,031, interquartile range [IQR]: 421-5767) (Fig. 1a). The lowest neutralizing activity (NAb50) was observed against HCV-JFH1 with HCV 1a (H77) envelope proteins (H77S/JFH1) (Fig. 1a).

**Table 1:** Patient characteristics

Characteristic	Patients with chronic HCV infection		Patients that received interferon-alpha-based treatment		Patients that received antiviral (DAA) treatment		Uninfected subjects
	serum neutralizing activity	B cell analysis	serum neutralizing activity	B cell analysis	serum neutralizing activity	B cell analysis	B cell analysis
*n*	24	48	17	5	7	43	17
Age, median (IQR), years	48 (39.5–55.8)	58 (49.3–65.8)	46.0 (38.8–49.6)	50 (47.5–50.0)	61.0 (47.1–63.9)	59 (51.2–65.4)	47.5 (40–59.8)
Sex, no. (% of male subjects)	12 (50.0%)	20 (41.7%)	8 (47.1%)	1 (20.0%)	4 (57.1%)	19 (44.1%)	8 (47.0%)
Race							
White, no. (%)	16 (66.7%)	33 (68.8%)	12 (70.6%)	5 (100%)	4 (57.1%)	28 (65.1%)	9 (52.9%)
Black, no. (%)	3 (12.5%)	9 (18.8%)	1 (5.9%)	0 (0.0%)	2 (28.6%)	9 (20.9%)	2 (11.8%)
Asian, no. (%)	5 (20.8%)	3 (6.3%)	4 (23.5%)	0 (0.0%)	1 (14.3%)	3 (7.0%)	1 (5.9%)
Other, no. (%)	0 (0.0%)	2 (4.2%)	0 (0.0%)	0 (0.0%)	0 (0.0%)	2 (4.7%)	2 (11.8%)
Unknown, no. (%)	0 (0.0%)	1 (2.1%)	0 (0.0%)	0 (0.0%)	0 (0.0%)	1 (2.3%)	3 (17.6%)
Clinical data prior to treatment							
HCV RNA (IQR), log10 IU/ml	6.5 (6.3–6.7)	6.1 (5.7–6.7)	detected, not quantitated	6.0 (5.4–6.7)	6.5 (6.3–6.7)	6.1 (5.7–6.7)	
HCV genotype							
1	12	38	5	4	7	34	
2	6	4	6	1	0	3	
3	6	4	6	0	0	4	
4	0	2	0	0	0	2	
ALT, median (IQR), IU/L	87 (53.5–153.5)	34 (26.0–63.8)	74 (53.0–156)	35 (32.0–35.0)	89 (69.5–117.5)	33 (26.0–64.5)	
HAI score, median (IQR), IU/L	10 (7.3–10.0)	5 (5.0–9.0)	10 (8.0–10.0)	9 (7.3–10.3)	8 (6.0–10.0)	5 (5.0–8.0)	
Fibrosis score							
0	3	7	2	0	1	7	
1	11	24	9	4	2	20	
2	0	0	0	0	0	0	
3	8	9	5	0	3	9	
4	2	3	1	0	1	3	

Interferon-alpha-based treatment consisted of either interferon-alpha or pegylated interferon-alpha plus ribavirin for 24, 36, or 48 weeks. Direct-acting antiviral (DAA) treatment consisted of either Sofobuvir + Velpatasvir (12 weeks) or Asunaprevir + Daclatasvir (24 weeks).

To assess how treatment-induced HCV clearance affects the serum neutralizing activity, we performed the same assays with serial serum samples that were collected during and after successful antiviral therapy (Fig. 1b and Supplementary Fig. 1a). All patients were repeatedly tested for HCV RNA and remained HCV RNA negative during the entire follow-up. Seventeen of the 24 patients underwent interferon-alpha-based therapy with follow-up of up to 18 years. Five of these patients were infected with HCV genotype 1, six with HCV genotype 2, and six with HCV genotype 3. The serum NAb50 was determined prior to and at up to five different time points during the 7–18 years of post-treatment follow-up. The decay of NAb50 was calculated based on a simple linear model of an exponential with the dependent variable normalized for each patient’s baseline value. As shown in Fig. 1b, the NAb50 significantly decreased for each of the HCVcc after successful therapy with a half time ranging from 4.8 to 7.4 years.

To exclude the possibility that the decrease in neutralizing activity was the result of immunomodulatory or immune-suppressive effects of the interferon-alpha-based treatment, we performed the same analysis during and after treatment with direct-acting antivirals (DAAs). Seven of the 24 patients with chronic HCV infection (Fig. 1a) underwent DAA-based therapy. All were infected with HCV genotype 1. Due to the relatively recent development of DAA, the follow-up time was a median of 3.8 years [IQR 2.9–4.4], thus shorter than for the patients who had received interferon-alpha-based therapy. Following the same approach as described above, the half-life in serum neutralizing activity was calculated from serial measurements of the NAb50 and ranged from 1.3 to 3.8 years (Fig. 1c).

Collectively, these results demonstrate that the NAb50 against all tested HCVcc decreases after successful interferon-alpha- or DAA-mediated elimination of HCV.

### HCV-neutralizing activity declines, whereas vaccine responses persist

**Fig. 2:**
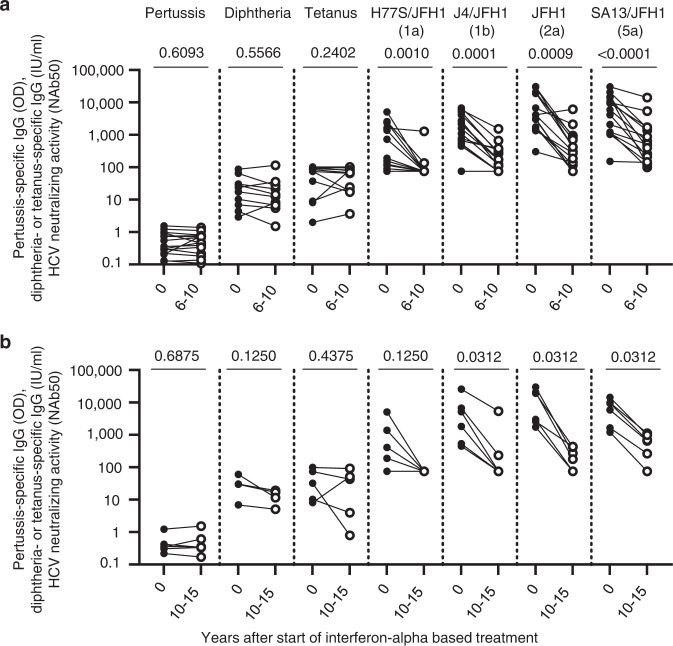
Patients with declining HCV-neutralizing activity maintain antibodies against pertussis, diphtheria, and tetanus toxoid antigens. **a**, **b** Humoral immune responses to pertussis, diphtheria, and tetanus toxoid antigens and HCV-neutralizing activity. Neutralization of HCVcc with JFH1 backbone and core-NS2 sequences of H77S (genotype 1a), J4 (genotype 1b), JFH1 (genotype 2a), and SA13 (genotype 5a) was assessed. Two-sided *p* values are shown and calculated using the Wilcoxon matched-pairs signed-rank test. OD, optical density. NAb50, 50% neutralizing activity. **a** Paired serum samples were studied prior to (filled circles) and 6–10 years after (open circles) start of interferon-alpha-based cure of chronic HCV infection (*n* = 15 sera tested against pertussis; *n* = 10 and *n* = 11 sera tested against diphtheria toxin and tetanus toxoid, respectively; *n* = 15 sera tested against each of the indicated HCVcc). **b** Paired serum samples were studied prior to (filled circles) and 10–15 years after (open circles) interferon-alpha-based cure of chronic HCV infection (*n* = 6 sera tested at each time point). Source data are provided as a Source Data file.

To assess whether the declining HCV-neutralizing activity represents a general immune phenotype in the patients or an effect of treatment, we compared the HCV-neutralizing activity with antibody titers against diphtheria, tetanus toxoid, and pertussis vaccine. As shown in Fig. 2a, patients maintained antibody titers to diphtheria, tetanus toxoid, and pertussis antigens even though their HCV-neutralizing activity declined 6–10 years after the cure of hepatitis C. The same pattern was observed when the pretreatment time point was compared to a time point 10–15 years after the cure of hepatitis C (Fig. 2b). Thus, the half-life of the HCV-neutralizing activity is much shorter than that of humoral responses against other antigens. This is consistent with the maintenance of antibodies against common viruses such as measles and mumps virus, which have been estimated to last for more than 200 years^38^.

### The breadth of HCV-neutralizing activity decreases after cure of chronic hepatitis C

To assess changes in the neutralization breadth after treatment-induced HCV clearance, we used a panel of 19 HCV pseudoparticles (HCVpp)^10,47^. This panel comprises 94% of the amino acid polymorphisms present at greater than 5% frequency in a reference panel of 643 HCV genotype 1 isolate from GenBank^10,48^. We have previously demonstrated that HCV genotype 1 HCVpp can be used to determine the neutralizing-breadth of plasma from patients irrespective of whether the latter are infected with HCV genotype 1, 2, or 3^12,49^. This is because the HCV genotype is determined by conserved sequences in the HCV 5’ untranslated, core or NS5 regions^50^, whereas neutralizing antibodies target the HCV E1E2 region. In fact, differences in neutralization sensitivity between HCVpps are independent of genetic distances between E1E2 clones^49^.

**Fig. 3:**
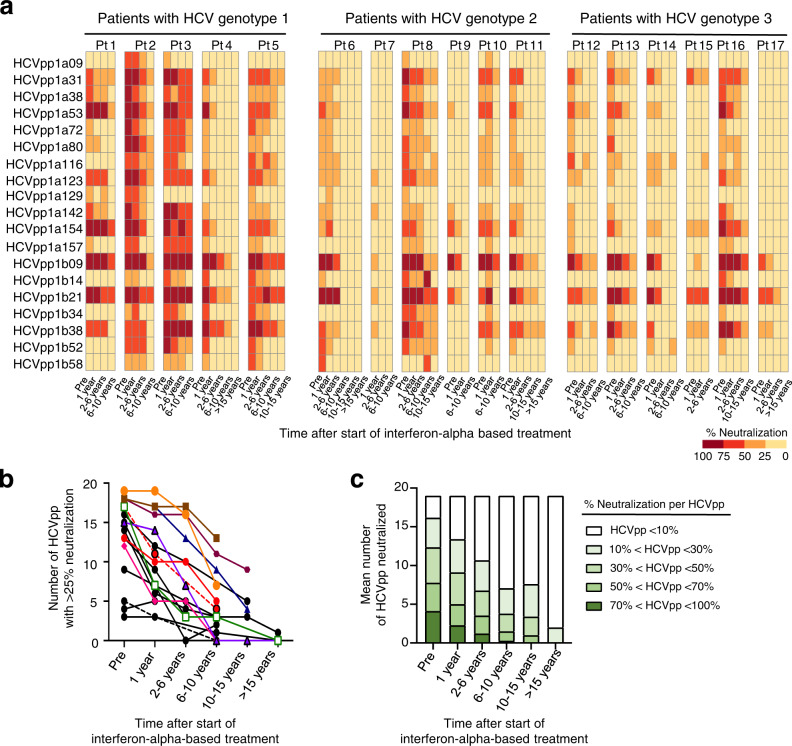
The breadth of serum neutralizing activity decreases after treatment-induced clearance of HCV. **a** The heat map represents the neutralizing activity of serially collected sera from patients (*n* = 17) at 4–6 time points prior to (pre) and after the start of interferon-alpha-based treatment of chronic HCV infection. Each serum was tested against a diverse panel of 19 HCVpp listed on the y-axis. The percentage of neutralization achieved by 1:100 dilution of serum, tested in duplicate, is indicated for each HCVpp. **b**, **c** Dynamic change in the neutralization breadth at serial time points prior to (pre) and after interferon-induced HCV clearance. **b** Number of HCVpp that were neutralized at >25% by the sera in (**a**). **c** Average number of HCVpp neutralized with the indicated neutralization efficacy by the sera in (**a**). Source data are provided as a Source Data file.

As shown in Fig. 3a, the serum neutralizing activity against each of the 19 HCVpp decreased after the patients had cleared HCV with interferon-alpha-based therapy. Likewise, the breadth of the neutralizing activity, as determined by the number of HCVpp with >25% neutralization, decreased for each patient (Fig. 3b). Likewise, the mean number of HCVpp with >70% neutralization decreased from 4.1 to 2.3 within a year of treatment, and more than half of the HCVpp were not neutralized at 6–10 years after treatment (Fig. 3c).

To assess the epitope specificities of NAbs present in the broadly neutralizing serum samples, we deconvoluted the results with a reference panel of 8 HCV-specific human monoclonal antibodies (mAb)^10^ that was previously tested against the same panel of 19 HCVpp^47,51^ (Table 2). Each of the 8 mAbs in this panel had a unique and reproducible neutralization profile against the 19 HCVpp, and functionally similar antibodies were clustered into groups^51^. One antibody (HEPC74) was included as a reference in neutralization assays to ensure that the antibody neutralization profile in the current study matched the previously published ones^10,47,51^.

**Table 2:** Deconvolution of NAbs in serum of patients after interferon-based treatment and clearance of chronic HCV infection

Pt	HCV genotype of patient	Year after start of treatment	Neutralization breadth [number of HCVpp neutralized]	Reference monoclonal antibodies								Statistics	
				AR1A	HC-1	HEPC98	CBH-2	AR3A	HEPC74	HC84.26	AR4A	Pearson	*P* value
1	1a	−0.24	13	0.23		0.16			0.25		0.27	0.8063	3.0 × 10^5^
		0.98	10			0.27			0.25		0.35	0.7946	4.8 × 10^5^
		4.82	10	0.2		0.14		0.12	0.28		0.25	0.7592	1.6 × 10^4^
		7.18	5	0.11		0.24					0.43	0.7433	2.6 × 10^4^
2	1a	−1.05	19	0.31		0.17		0.11	0.16		0.25	0.769	1.1 × 10^4^
		0.92	19	0.37					0.28		0.25	0.8515	3.7 × 10^6^
		3.67	16			0.18		0.11	0.11		0.55	0.6328	3.6 × 10^3^
		7.06	7			0.31					0.54	0.7515	2.0 × 10^4^
3	1a	−0.21	18					0.34			0.49	0.7659	1.3 ×10^4^
		0.93	17			0.15		0.16			0.6	0.8296	1.1 × 10^5^
		3.28	17			0.24		0.17	0.11		0.48	0.8185	1.8 × 10^5^
		9.25	13			0.2		0.19	0.25		0.29	0.7819	7.6 × 10^5^
4	1a	−1.97	17				0.11		0.35	0.27	0.21	0.9199	2.5 × 10^8^
		0.98	7			0.11		0.16	0.28		0.41	0.8367	7.9 × 10^6^
5	1b	−0.98	18				0.14			0.26	0.49	0.8461	5.0 × 10^6^
		0.92	17			0.14	0.12			0.31	0.26	0.871	1.2 × 10^6^
		4.95	13			0.22	0.22				0.55	0.7394	2.9 × 10^4^
		9.11	9			0.23	0.17				0.54	0.7576	1.7 × 10^4^
6	2	−1.00	15						0.51	0.14	0.35	0.5702	1.0 × 10^2^
		0.99	14	0.12		0.19			0.11		0.5	0.6886	1.1 × 10^3^
		5.83	7			0.31					0.59	0.6927	1.0 × 10^3^
7	2b	0.24	12			0.23	0.16				0.57	0.6611	2.0 × 10^3^
		0.96	5								0.84	0.7857	6.7 × 10^5^
		2.92	5					0.34		0.11	0.55	0.6179	4.8 × 10^3^
8	2b	−1.09	18					0.18	0.22	0.22	0.35	0.8558	2.9 × 10^6^
		1.11	16						0.16	0.26	0.34	0.8975	1.8 × 10^7^
		4.19	16			0.14		0.2	0.16		0.42	0.8008	3.8 × 10^5^
		9.75	11				0.29		0.5		0.21	0.5249	2.1 × 10^2^
		13.64	9		0.17		0.19		0.18		0.43	0.8026	3.5 × 10^5^
9	2a	−1.09	5			0.15			0.27		0.5	0.7498	2.1 × 10^4^
10	2b	−1.07	17	0.11		0.28			0.21		0.3	0.789	5.9 × 10^5^
		0.93	11			0.26		0.12			0.62	0.751	2.1 × 10^4^
		9.27	4			0.29		0.22			0.38	0.7417	2.7 × 10^4^
11	2a	−0.12	13			0.23		0.18			0.53	0.7519	2.0 × 10^4^
		1.00	9			0.15					0.77	0.7044	7.6 × 10^4^
12	3b	−0.05	14			0.24					0.56	0.7135	6.0 × 10^4^
		1.10	6			0.29			0.15		0.41	0.7833	7.2 × 10^5^
		3.84	4			0.36					0.48	0.711	6.4 × 10^4^
13	3b	−0.46	15		0.13	0.13		0.25	0.32		0.17	0.7497	2.1 × 10^4^
		0.88	7		0.12	0.25			0.19		0.29	0.7521	2.0 × 10^4^
		4.41	5			0.25					0.54	0.6574	2.2 × 10^3^
14	3a	−2.20	9			0.12				0.16	0.55	0.7228	4.7 × 10^4^
		1.10	7			0.22					0.65	0.6728	1.6 × 10^3^
15	3a	0.92	5		0.13	0.39				0.11	0.32	0.7322	3.6 × 10^4^
		2.26	5		0.14	0.4					0.41	0.7754	9.6 × 10^5^
16	3a	−0.39	16			0.11		0.13	0.35		0.25	0.8744	9.8 × 10^7^
		0.90	12			0.25		0.12	0.25		0.32	0.8278	1.2 × 10^5^
		5.73	10			0.29		0.13			0.45	0.7591	1.6 × 10^4^
		13.52	5			0.25			0.15		0.41	0.736	3.2 × 10^4^

Values are the proportion of each serum neutralization response attributed to each reference mAb, using a previously described deconvolution approach (10). Proportions greater than 0.1 are shown. The neutralization breadth is the number of 19 HCVpp that are neutralized greater than 50% by a 1:100 dilution of serum. *P* values are for Pearson correlation between serum sample neutralization profile and the best-fit combined reference mAb neutralization profile.

For deconvolution analysis, we used an algorithm that identifies a reference mAb neutralization profile with a minimum difference from the serum neutralization profile (GitHub repository: https://github.com/BaileyLabHCV/Neutralizing-breadth.git)^10^. This approach allowed us to calculate the proportion with which each reference mAb contributed to the neutralization profile of each sample. The neutralization profile of each mAb was converted to scaled neutralization profiles by multiplying the proportions determined by the deconvolution. Thereafter, the scaled neutralization profiles of all reference mAb were added to generate a combined neutralization profile. Then, the quality of fit of each serum was examined by comparing this combined neutralization profile with the serum neutralization profile using Pearson’s correlation.

Consistent with previous results of patients who clear acute HCV infection or have chronic HCV infection^10^, the majority of HCV-neutralizing activity in the serum was attributed to the set of reference mAbs with known epitope specificities. Prior to treatment, the neutralization response of most patients’ sera was comparable to the neutralization response of four mAb from the reference panel: HEPC98, AR3A, HepC74, and AR4A (Table 2). The latter three mAb are known for their broad neutralization profile. Notably, pretreatment sera from all patients had AR4A-like neutralizing activity. This AR4A-like neutralizing activity remained a large proportion of the total neutralizing activity after treatment-induced HCV clearance.

Collectively, the results show a decrease in the serum neutralizing activity after treatment-induced HCV clearance with relative preservation of specific mAb-types.

### The differential phenotype of HCV-specific and total memory B cells in HCV-infected patients and uninfected controls

**Fig. 4:**
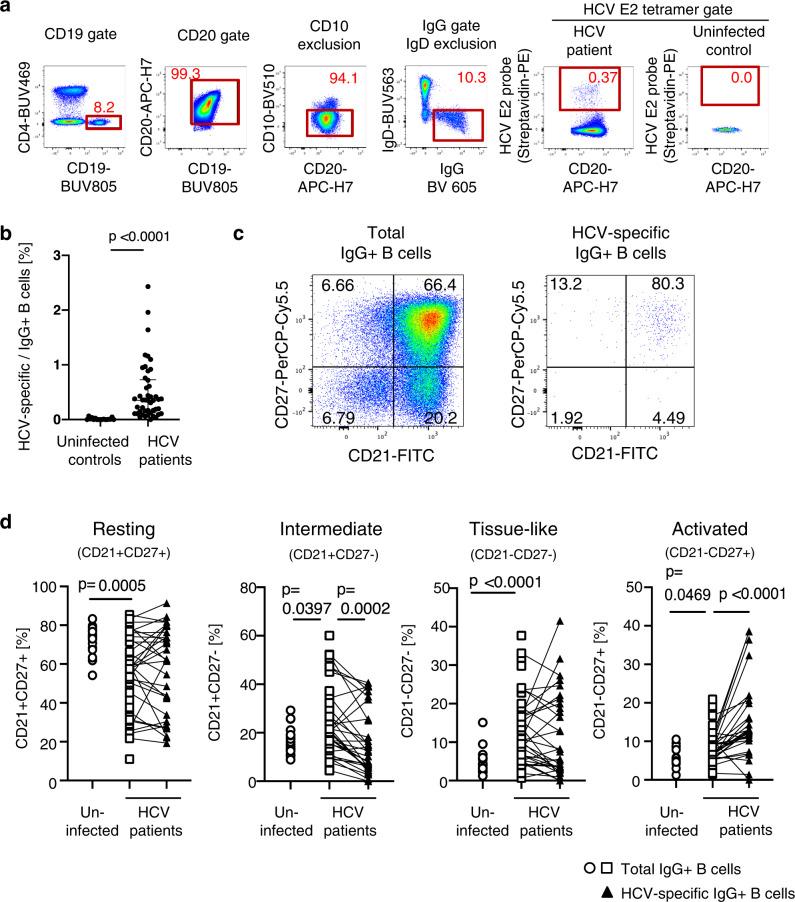
Differential phenotype of HCV-specific and total memory B cells in HCV-infected patients and uninfected controls. **a** Representative flow cytometry plots illustrating the gating strategy to identify HCV envelope 2 (E2)-specific memory B cells. **b** Frequency of HCV-specific B cells in the blood of patients with chronic HCV infection (*n* = 48) and uninfected controls (*n* = 17). Each data point represents one study participant; median + IQR are shown. Two-sided *p* values were calculated with the Mann–Whitney *U*-test. **c** Representative flow cytometry plots illustrating CD10- (mature) IgG+ (class-switched) total memory B cells and HCV-specific B cells examined for CD21 and CD27 expression. **d** Size of B cell subsets with a resting, intermediate, tissue-like, and activated phenotype among total or HCV-specific IgG+CD10- peripheral blood B cells of patients with chronic HCV infection (*n* = 48 and *n* = 31, respectively) and uninfected controls (*n* = 17). Samples with less than 30 HCV E2 tetramer+ events were excluded from CD21/CD27 subset analysis. Two-sided *p* values were calculated using the Wilcoxon signed-rank test was used for paired data or the Mann–Whitney *U*-test for unpaired data. Source data are provided as a Source Data file.

Next, we set out to characterize HCV-specific memory B cells by flow cytometry. Using a biotin-conjugated HCV E2 probe^52^, we detected HCV-specific memory B cells in the CD10- (mature) IgG + (class-switched) B cell population in the blood of patients with chronic HCV infection but not in uninfected controls (Fig. 4a, b).

Based on CD21^53^ and CD27 expression^54,55^, we compared the composition of the CD10- IgG+ B cell population of chronically HCV-infected patients and uninfected controls (Fig. 4c). For this analysis, we first studied the total mature B cell population irrespective of its antigen specificity in chronic HCV infection. CD21 + CD27 + resting memory B cells constituted the largest subset but were slightly less frequent in the blood of patients with chronic HCV infection than in uninfected controls (median: 58%, IQR: 34–73% versus median: 74%, IQR: 68–77%, *p* = 0.0005, Mann–Whitney test) (Fig. 3d, left panel). In contrast, the CD21+CD27- intermediate, the CD21-CD27- tissue-like and the CD21-CD27+ activated memory B cell subsets were expanded in the blood of patients with chronic HCV infection compared to uninfected controls (intermediate memory; 21% [16–34% IQR] vs 16% [13–20% IQR], *p* = 0.0397, tissue-like memory; 10% [5–18% IQR] vs 4% [2–6% IQR], *p* < 0.0001, activated memory; 8% [6–10% IQR] vs 6% [5–9% IQR], *p* = 0.0469, Mann–Whitney test) (Fig. 4d, middle and right panels).

Next, we studied the composition of the HCV-specific B cell population. We found that the HCV-specific mature B cell population was significantly enriched for activated (CD21-CD27+) memory B cells in chronically HCV-infected patients (median: 13%, IQR: 10–20%) compared to 7% [6–9%] in the total B cell population, *p* < 0.0001, Wilcoxon signed-rank test). In contrast, it contained fewer B cells with an intermediate memory phenotype than the total B cell population (median: 12%, IQR: 6–26% vs median: 21%, IQR: 13–30%, *p* = 0.0002, Wilcoxon signed-rank test) (Fig. 4d).

Collectively, the results show that the phenotype of HCV-specific memory B cells was distinct from that of the total mature IgG+ memory B cell population in chronic HCV infection.

### HCV-specific memory B cells express liver-homing chemokine receptors

**Fig. 5:**
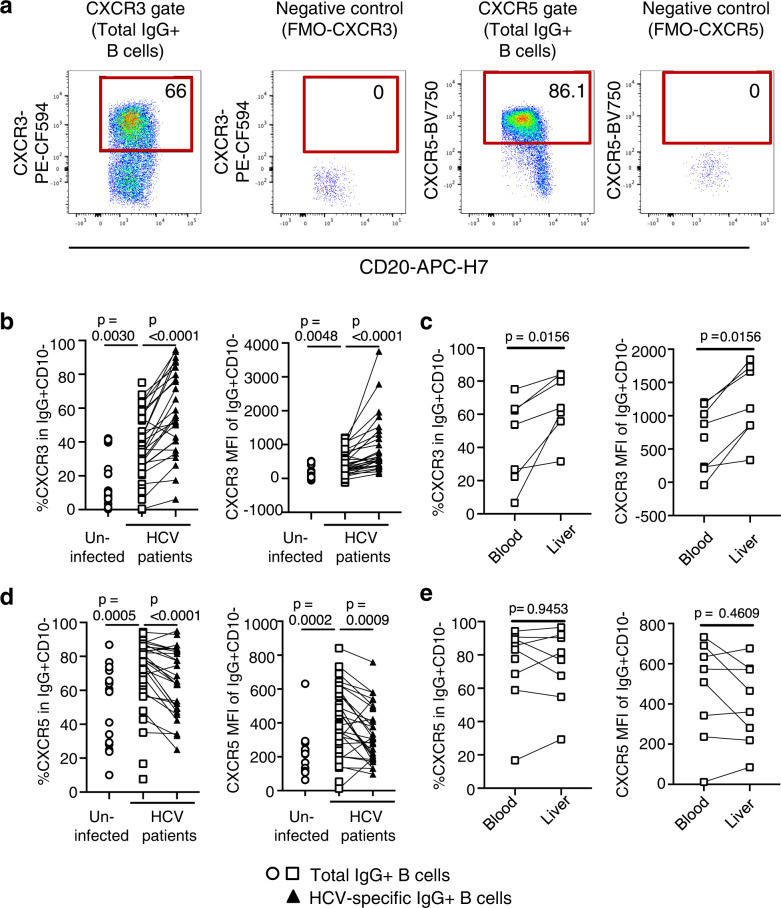
HCV-specific memory B cells express a liver-homing chemokine receptor profile. **a** Representative flow cytometry plots illustrating CXCR3 and CXCR5 expression on IgG+ B cells. FMO, fluorescence-minus-one. **b** The frequencies of CXCR3+ (liver-homing) IgG+ B cells from patients with chronic HCV infection (*n* = 42) and uninfected controls (*n* = 17), and of CXCR3+ HCV-specific IgG+ B cells from patients with chronic HCV infection (*n* = 27) were compared (left panel). The CXCR3 geometric mean fluorescent intensity (MFI) of IgG+ B cells from patients with chronic HCV infection (*n* = 42) and uninfected controls (*n* = 17), and of HCV-specific IgG+ B cells from patients with chronic HCV infection (*n* = 29) were compared (right panel). **c** The frequencies of CXCR3+ (liver-homing) B cells (left panel) and their CXCR3 MFI (right panel) were compared in paired blood and liver specimens of patients with chronic HCV infection (*n* = 7 paired samples in left panel, *n* = 8 in right panel). **d** The frequencies of CXCR5+ (lymph node-homing) IgG+ B cells from patients with chronic HCV infection (*n* = 42) and uninfected controls (*n* = 17), and of CXCR5+ HCV-specific IgG+ B cells from patients with chronic HCV infection (*n* = 27) were compared (left panel). The CXCR5+ MFI of IgG+ B cells from patients with chronic HCV infection (*n* = 42) and uninfected controls (*n* = 17), and of HCV-specific IgG+ B cells from patients with chronic HCV infection (*n* = 28) were compared (right panel). **e** The frequencies of CXCR5+ (lymph node-homing) B cells (left panel) and their CXCR5 MFI (right panel) were compared in paired blood and liver specimens of patients with chronic HCV infection (*n* = 8 paired samples in each panel). Two-sided *p* values were calculated with the Wilcoxon signed-rank test for paired data and the Mann–Whitney *U*-test for unpaired data. Each data point represents one study participant. Samples with less than 30 HCV E2 tetramer+ events were excluded (**b**–**e**). Source data are provided as a Source Data file.

The total mature B cell population of patients with HCV infection was enriched for cells that expressed the chemokine receptor CXCR3 (Fig. 5a) when compared to the corresponding mature B cell population of uninfected controls (Fig. 5b, left panel). This was recapitulated by a higher CXCR3 geometric mean fluorescence intensity (MFI) on B cells of patients with HCV infection compared to those of uninfected controls (Median 69 [3–312 IQR] vs 336 [179–608 IQR], *p* = 0.0048, Mann–Whitney test) (Fig. 5b, right panel). Both the percentage of CXCR3+ B cells and the CXCR3 MFI were even higher for HCV-specific B cells than for the total B cell population (% CXCR3+ : median 55% [39–81%] vs 38% [27–55% IQR], *p* < 0.0001; CXCR3 MFI: median 592 [387–1266 IQR] vs 348 [232–708 IQR], *p* < 0.0001, Wilcoxon signed-rank test, Fig. 5b). This finding extended to all of the four CD21/CD27 subsets (Supplementary Fig. 2a, b). CXCR3 promotes homing to inflammatory sites, such as the HCV-infected, inflamed liver. Consistent with the function of CXCR3 as a liver-homing chemokine receptor, the percentage of CXCR3+ cells and the CXCR3 MFI were significantly higher in the B cell population in the liver than in the blood (% CXCR3+ : median 64% [56–84% IQR] vs 54% [22–63% IQR], *p* = 0.0156; CXCR3 MFI: median 1109 [856–1732 IQR] vs 879 [210–1180 IQR], *p* = 0.0156, Wilcoxon signed-rank test) (Fig. 5c).

Interestingly, the total mature B cell population of patients with HCV infection was also enriched for cells that expressed the lymph node-homing chemokine receptor CXCR5 (Fig. 5a) when compared to the corresponding mature B cell population of uninfected controls (Fig. 5d, left panel), and this difference held true when the CXCR5 MFI was examined (CXCR5; median 218 [114–264 IQR] vs 396 [267–573 IQR], *p* < 0.0001) (Fig. 5d, right panel). However, both the percentage of CXCR5 + cells and the CXCR5 MFI were significantly lower for HCV-specific B cells than for total mature B cells (% CXCR5+; *p* < 0.0001 and CXCR5 MFI; *p* = 0.0009, Fig. 5d). When HCV-specific and total mature B cells were separated in subsets based on CD21 and CD27 expression, this difference in the percentage of CXCR5+ cells and in CXCR5 MFI was only observed in the respective resting (CD21+CD27+ ) subset (Supplementary Fig. 2c, d). Consistent with the function of CXCR5 as a lymph node-homing chemokine receptor, there was no enrichment for CXCR5+ B cells in the liver (Fig. 5e).

Collectively, these results demonstrate that the phenotype of HCV-specific memory B cells is distinct from that of total IgG+ memory B cells, with increased expression of the liver-homing marker CXCR3 on all CD21/CD27 subsets and decreased expression of the lymph node-homing marker CXCR5 on the CD21+CD27+ (resting) B cell subset.

### Phenotype of HCV-specific memory B cells after cure of chronic hepatitis C

We then investigated how the HCV-specific memory B cell population changes after treatment-induced HCV clearance. We selected ten DAA-treated and four interferon-alpha-treated patients with chronic HCV infection based on the following three criteria: (i) greater than 3 years of follow-up after successful DAA treatment or greater than 10 years of follow-up after successful interferon-alpha-based treatment, (ii) greater than 30 HCV-specific memory B cells detectable per 10 million PBMC at the pretreatment time point, and (iii) available cryopreserved PBMC at multiple time points prior to and after HCV clearance.

**Fig. 6:**
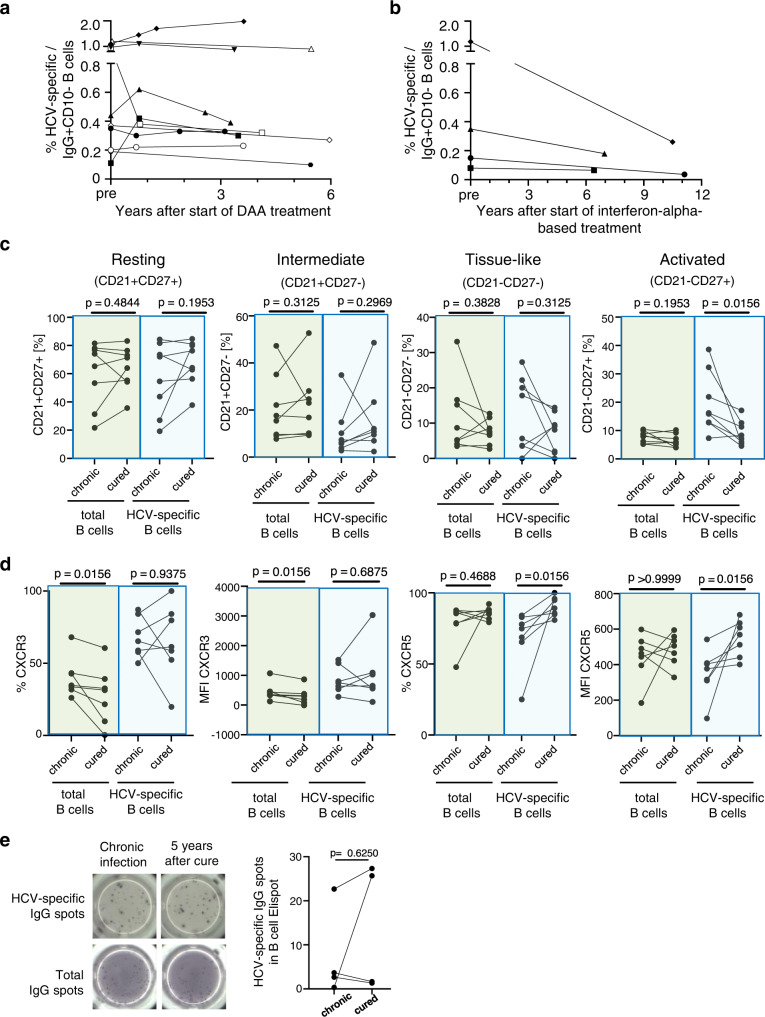
Phenotype of HCV-specific memory B cells after cure of chronic hepatitis C. **a**, **b** Longitudinal change in the frequency of HCV-specific B cells prior to and after DAA- (**a**) or interferon-alpha-based cure of chronic hepatitis C (**b**). *n* = 10 patients were studied at 2–5 time points each (**a**); *n* = 4 patients were studied at two time points each in (**b**). **c** Differential frequency of CD21/CD27 B cell subsets within the HCV-specific and total IgG+ B cell population prior to and >3 years after HCV clearance. Paired samples from *n* = 8 patients were studied. **d** The frequency of CXCR5+ and CXCR3+ B cells and the MFI of CXCR5 or CXCR3 expression within the HCV-specific and IgG+ B cells populations prior to and >3 years after HCV clearance. Paired samples from *n* = 7 patients were studied. **e** B cells from paired PBMC samples prior to and after treatment-induced HCV clearance were differentiated into antibody-secreting cells (ASCs). The number of ASCs that secrete HCV-specific and total IgG was determined by Elispot analysis. Paired samples from *n* = 4 patients were studied. Two-sided *p* values were determined with the Wilcoxon matched-pairs signed-rank test. Source data are provided as a Source Data file.

As shown in Fig. 6a, the frequency of HCV-specific memory B cells was relatively stable for at least 6 years after DAA-induced HCV clearance. As interferon-alpha-based therapy preceded DAAs as a state-of-the-art treatment for HCV, this allowed us to investigate changes in the frequency of HCV-specific memory B cells over an even longer period. As shown in Fig. 6b, HCV-specific B cells remained detectable in the circulation even 10 years after treatment-induced HCV clearance.

Next, we evaluated the subset composition of HCV-specific and total memory B cells in patients with at least 3 years of follow-up after treatment-induced HCV clearance (range: 3 to 10 years). Whereas the CD21/CD27 subsets of the total B cell population were not affected by HCV clearance, there was a significant decrease in the percentage of activated (CD21-CD27+) cells in the HCV-specific memory B cell population (median 16% [13–30% IQR] vs median 8% [6–12% IQR], *p* = 0.0156 (Fig. 6c). This was accompanied by an increase in the frequency of HCV-specific B cells that expressed the lymph node-homing marker CXCR5 (median 75% [65–83% IQR] vs median 89% [85–96% IQR], *p* = 0.0156) and an increase in the CXCR5 MFI on these cells (median 377 [310–409 IQR] vs median 569 [441–634 IQR] *p* = 0.0156). No such change was observed for the total IgG+ memory B cell population (Fig. 6d).

Collectively, these data indicate that treatment-induced clearance of HCV and removal of HCV antigen reduces the activation of HCV-specific B cells. The increase in CXCR5 expression may route them to lymph nodes after HCV is cleared from the liver.

### Intact function of HCV-specific memory B cells after cure of hepatitis C

Finally, we assessed the functional capacity of HCV-specific B cells prior to and after treatment-induced HCV clearance. We magnetically sorted B cells from paired PBMC samples that were cryopreserved prior to or post-DAA treatment and used an in vitro stimulation protocol to evaluate their capacity to differentiate into antibody-secreting cells (Fig. 6e). The number of HCV-specific B cells with antibody-secreting capacity was determined in an Elispot assay, in which the cells were seeded onto anti-IgG-coated plates. B cell-secreted IgG was captured by plate-bound anti-IgG and the specificity of the secreted IgG was determined by staining with biotin-conjugated HCV E2 protein (Fig. 6e). A comparison of HCV-specific B cell spots from paired PBMC samples prior to and post-DAA-treatment (median 5.5 years [4.1–5.8 years IQR]) demonstrated a preserved capacity to differentiate into HCV-specific antibody-secreting cells. Collectively, these results indicate that the capacity of B cells to differentiate into HCV-specific antibody-producing cells is maintained after treatment-induced HCV clearance.

## Discussion

Studying a unique set of serially collected and cryopreserved sera and PBMC of HCV patients who were successfully treated for HCV, we found a rapid decline of serum neutralizing activity against 4 HCVcc. Using a panel of 19 genotype-1 HCVpp, we also observed a reduction in neutralization breadth, as indicated by the number of HCVpp neutralized. This HCVpp panel was used because it is antigenically diverse, and because the deconvolution algorithm had been optimized for this panel^10^. HCVpp in this panel span Tiers 1–3 of neutralization resistance, as defined by a more recently developed multi-genotype HCVpp panel spanning four tiers of increasing neutralization resistance^49,56^. We have also demonstrated that the HCV genotype of the infected patient does not affect the neutralization breadth of the measured with the genotype 1 panel^12^, and that the neutralization breadth measured with the genotype 1 panel correlates with the neutralization breadth measured with the multi-genotype panel^49,56^.

Interestingly, the deconvolution of the serum neutralizing activity revealed the preservation of an AR3- and AR4A-like profile after clearance of chronic HCV infection that has also been described after clearance of acute HCV infection^10,26^. These results indicate that broadly neutralizing antibodies persist from the acute phase through decades of chronic HCV infection. Thus, patients who clear HCV infection spontaneously and patients who clear via treatment can maintain a qualitatively similar neutralizing activity^12,13^.

Why does HCV-neutralizing activity decrease after the cure of chronic hepatitis C? Generally, antibodies are produced by two main B cell populations, long-lived plasma cells, and memory B cells^35^. Long-lived plasma cells secrete highly selected and specific antibodies, and in other infections, are known to maintain serum antibody levels independently from memory B cells^57^. In contrast, memory B cells secrete antibodies with less avidity but a broader range, thus allowing recognition of pathogens with variant sequences^35^. After antigen recognition, memory B cells can differentiate into plasma cells or re-enter the germinal centers to replenish the resident memory B cells. During chronic infection, memory B cells are continuously stimulated by HCV antigens and differentiate into antibody-producing cells. We have found that HCV E2-specific memory B cells have a distinct profile compared to total memory B cells during chronic HCV infection. Despite the declining neutralizing activity in the serum, they persist in circulation up to 10 years after HCV clearance.

While our protocol did not detect the subset of B cells that recognizes epitopes that bridge E1 and E2, it should be noted that the majority of known broadly neutralizing antibodies are E2-specific and thus detected in our assays. Consistent with our finding, Merat et al. reported that B cell clones derived 25 years after spontaneous HCV clearance from people who inject drugs, secrete broadly neutralizing HCV-specific antibodies^58^. Likewise, Olbrich et al. cloned two antibodies against a linear E2 epitope from memory B cells that were isolated more than 30 years after spontaneous HCV clearance^59^. Thus, it is likely that HCV-specific memory B cells are long-lived after HCV clearance, regardless of whether HCV clearance is achieved spontaneously after acute infection or, as in our study, induced by treatment of chronic infection. The reduction in serum neutralization strength despite the persistence of memory B cells suggests that long-lived plasma cells have a minor contribution to antibody production in chronic HCV infection.

What can be the reasons for the impaired generation of HCV-antibody-producing long-lived plasma cells in HCV infection? A key feature of acute HCV infection is the rapid induction of type I and III IFN, which results in high levels of ISG expression^60^. Type I IFN is known to inhibit the generation of long-lived plasma cells through depletion of antiviral B cells and through modulation of CD8 + T cells and inflammatory monocyte responses^61–63^. Other important factors to be taken into account are the formation of germinal centers and the presence of follicular T cells (Tfh). In germinal centers, B cells undergo clonal expansion, class switching, and somatic hypermutation and are selected by Tfh cells to differentiate into memory B cells and long-lived plasma cells. If the quality of the T cell - B cell interaction at the T-B cell border of the follicle is inadequate, B cells are not induced to enter the germinal center but rather proliferate and differentiate into short-lived antibody-secreting plasma cells^43,61–63^. Indeed, the lack of early Tfh cell response and the reduced activation of HCV-specific memory B cells has recently been described in chronically evolving acute HCV infection^64^. Tfh cells remain reduced in number and functionally impaired during chronic HCV infection, as shown by the skewing of CD4 + T cells to a Th1 rather than Tfh phenotype. In mouse models, this defect in Tfh cell differentiation is associated with a subsequent defect of the germinal center response. It can be restored by the blockade of proinflammatory cytokines such as TNF-α and IFN-γ ^65,66^.

Thus, a prolonged inflammatory response appears to contribute to impaired Tfh cell differentiation and germinal center loss, resulting in the failure to generate long-lived plasma cells to maintain serum neutralizing activity. In this situation, the persistence of HCV-specific memory B cells is notable. Their ability to differentiate into antibody-secreting cells provides an opportunity to enhance the neutralizing antibody response by boosting HCV-specific memory B cells with candidate vaccines. The preserved response against the AR4A epitope may serve as a starting point for the development of vaccines. The treatment-induced elimination of inflammation^67^ and the improved Tfh cell phenotype after virus elimination^68^ should allow concomitant Tfh cell activation and—via appropriate interaction between memory B cells and Tfh cells—the generation of long-lived plasma cells. This can be aided by the use of new adjuvants that are being developed^69^.

In conclusion, our findings demonstrate that serum neutralizing activity after HCV infection is short-lived even though HCV-specific memory B cells are maintained. In addition to boosting HCV-specific memory B cells, the generation of germinal centers with appropriate interaction between memory B cells and Tfh cells will be important for a successful HCV vaccine that induces protective immunity in these patients.

## Methods

### Patients and biosamples

Cryopreserved sera and PBMC were selected from NCT00001729^70^, NCT00028093^71^, NCT02468648^72^, and NCT01888900^73^ based on the number of samples available and/or the length of follow-up after treatment-induced clearance of HCV infection. Patients were treated either with interferon-alpha-based regimens or with direct-acting antivirals (asunaprevir/daclatasvir or sofosbuvir/velpatasvir combination therapy) under these protocols. We also used cryopreserved sera and PBMC from NCT00001971, which is not a clinical intervention protocol but a protocol that allows the collection of biosamples for immunological analysis from patients with current or past liver disease. In addition, PBMC, sera, and liver biopsies were collected under NCT03520660. Recruitment for this trial is still ongoing, and we are reporting baseline data (before the start of antiviral therapy of patients that were recruited to the treatment phase of the trial).

All patients were followed at the NIH clinical center, which allowed us to exclude re-exposure and injection drug use (Table 1). Sera and PBMCs from uninfected blood donors obtained from the Department of Transfusion Medicine, NIH were studied for comparison. Protocols were approved by the Institutional Review Board of the National Institute of Diabetes and Digestive and Kidney Diseases (NIDDK) and the National Institute of Arthritis and Musculoskeletal and Skin Diseases (NIAMS), and patients gave written informed consent for research testing.

### Cell Lines

Huh7.5.1 hepatoma cells were generated and kindly provided by Dr. Francis V. Chisari, The Scripps Research Institute, La Jolla, CA^74^. They were maintained in Dulbecco’s modified Eagles medium (DMEM, Corning, New York, NY) supplemented with 10% fetal bovine serum (FBS, Serum Source International, Charlotte, NC), 1% penicillin/streptomycin, and 2 mM l-glutamine (Mediatech, Manassas, VA).

HEK293T cells and Hep3B cells were kindly provided by Dr. Justin Bailey (John Hopkins University School of Medicine, Baltimore, MD) and originally purchased from ATCC (catalog numbers CRL-3216 and HB-8084, respectively). HEK293T cells were maintained in DMEM, 10% FBS, 2 mM l-glutamine, 1x non-essential amino acids (Sigma-Aldrich, St. Louis, MO), and 1 mM sodium pyruvate (Thermo Fisher Scientific, Waltham, MA). HEP3B hepatoma cells were maintained in Eagle’s minimal essential medium (MEM, Corning) supplemented with 10% FBS, 2 mM l-glutamine, 1x non-essential amino acids, 1 mM sodium pyruvate, and 1% penicillin/streptomycin.

### Neutralization assays

Patient sera were evaluated for neutralization of cell culture-derived HCV (HCVcc) and HCV pseudoparticles (HCVpp).

Neutralization of HCVcc: HCVcc stocks were prepared from plasmids H77S/JFH1 (genotype 1a, accession no. EU363761); J4/JFH1 (genotype 1b, accession no. FJ230881), SA13/JFH1(genotype 5a, accession no. FJ393024), kindly provided by Dr. Jens Bukh (University of Copenhagen, Denmark), and JFH1 (2a, accession no. AB047639), kindly provided by Dr. Takaji Wakita (National Institutes of Infectious Diseases, Japan)^74–76^. The indicated genotypes and accession numbers refer to the core-NS2 sequences of the HCVcc. Each plasmid was digested with XbaI (New England Biolabs, Ipswich, MA). Linear RNA served as a template for in vitro transcription with MEGAscript T7 Kit (Thermo Fisher Scientific). Ten micrograms in vitro transcribed HCV RNA were transfected into 10^7^ Huh7.5.1 cells using DMRIE-C (Thermo Fisher Scientific) and Opti‑MEM I Reduced Serum Medium (Thermo Fisher Scientific). Supernatants were collected 4 to 6 days later and stored at −80 °C.

For HCVcc neutralization assays^77^, heat-inactivated serum samples were serially diluted from 1:25 to 1:25,600 and mixed with HCVcc for 1 h at 37 °C. Thereafter, the respective mixes were added to Huh7.5.1 cells that had been incubated overnight at 37 °C at 9000 cells/well in poly-d-lysine coated 96-well plates (Thermo Fisher Scientific). After 3 h incubation at 37 °C, the cell culture medium was replaced with a new medium and the plates were returned to 37 °C for an additional 48 h. Subsequently, the cells were fixed with 4% formaldehyde for 10 min and incubated for 30 min at room temperature with a blocking buffer that consisted of 5% normal goat serum, Vector Laboratories, Burlingame, CA), 0.3% TritonX-100 (Sigma-Aldrich), and 3% bovine serum albumin (MP Biomedicals, Solon, OH). Cells were subsequently incubated with 3% H_2_O_2_ (Sigma-Aldrich) for 5 min, stained with an HCVcore-specific mAb (#1868, 1:500, ViroStat, Westbrook ME) and goat anti-human/mouse-HRP (1:300, Jackson Immuno Research Lab, West Grove, PA) and developed with 3,3′Diaminobenzidine substrate (Agilent, Santa Clara, CA). The number of focus-forming units (FFU) was determined using an ImmunoSpot reader with BioSpot 5.0 software (Cellular Technology Limited, Shaker Heights, OH). FFU was converted to %FFU based on the FFU in the wells that had received HCVcc without serum. The 50% neutralization antibody titer (NAb50) was determined by non-linear regression using a sigmoidal fit model (Prism ver8.0.1, GraphPad Software, CA).

Neutralization of HCVpp: A panel of 19 HCV genotype 1 pseudoparticles was produced by transfecting the respective HCV E1/E2 plasmid (1a09, 1a31, 1a38, 1a53, 1a72, 1a80, 1a116, 1a123, 1a129, 1a142, 1a154, 1a157, 1b09, 1b14, 1b21, 1b34, 1b38, 1b52, and 1b58), the pNL4-3.Luc.R-E, and the pAdVantage plasmid into HEK293T cells^32,51,78^. All plasmids were kindly provided by Dr. Justin Bailey (Johns Hopkins University). For HCV neutralization assays^10,79^, heat-inactivated serum samples at 1:100 dilution or mAbs at 50 μg/mL were incubated with HCVpp for 1 h at 37 °C. Thereafter, the respective mixes were added to triplicate wells of HEP3B cells that had been seeded at 10,000 cells/well in 96-white plates the prior day. After a 5-h incubation at 37 °C, the cell culture medium was replaced with a new medium and the plates were returned to 37 °C for additional 72 h incubation. Luciferase activity was determined in cell lysates in relative light units (RLUs) using a CLARIOstar Plus microplate reader (BMG LABTECH, Cary, NC). Consistent with a prior report^10^, only HCVpp with RLU values >10^6^ were used in neutralization assays, and the HCVpp concentration produced values between 1 × 10^6^ and 6 × 10^6^ RLU. HEPC74, kindly provided by Dr. Bailey, Johns Hopkins University, was used as a positive control. Human IgG (catalog number 4506-10MG, Sigma-Aldrich, 1:20 dilution) was used as negative control. Percent neutralization was calculated as (1-RLU in the presence of serum/RLU in the presence of IgG) × 100. Percent neutralization was converted to the rank order of 19 HCVpp, generating a serum neutralization profile for each serum sample for the deconvolution algorithm.

To further analyze the neutralization capacity of each patient’s serum, deconvolution was performed^10,26^ using published neutralization profiles for reference mAbs (HC84.26, HC-1, CBH-2, AR1A, AR3A, AR4A, HEPC74, and HEPC98)^10^. A reference profile was generated for each serum sample. A scaled neutralization profile for each reference mAb was calculated by multiplying the neutralization profile of that reference mAb by the proportion of that mAb type (range, 0–1) calculated to be present in the serum sample. Eight scaled reference mAb neutralization profiles were added to generate a combined reference mAb neutralization profile, which was then compared to the actual serum neutralization profile by Pearson’s correlation. NAb deconvolution for any serum sample was considered a good fit if the correlation between the combined mAb neutralization profile and the serum neutralization profile had a *p* value less than 0.05 (two-sided test, Pearson). This deconvolution was applied in samples that neutralized at least 4 HCVpps by >25%, and only deconvolution proportions greater than 0.1 were considered to be significant.

### Quantitation of DTaP antigen-specific IgG

Plasma IgG antibodies against diphtheria, tetanus toxoid, and pertussis (DTaP vaccine) antigens were quantitated using Diphtheria IgG ELISA, Tetanus Toxoid IgG ELISA, and Bordetella pertussis IgG ELISA (Immuno-Biological Laboratories, Inc., Minneapolis, MN), respectively, according to the manufacturer’s instruction. Patients with evidence of booster vaccination were excluded.

### Isolation of mononuclear cells from liver biopsies and blood

Liver biopsy specimens from HCV-infected patients were suspended in Roswell Park Memorial Institute (RPMI)1640 medium (Mediatech) with 10% fetal bovine serum, 1% penicillin/streptomycin, 2 mM l-glutamine, and 10 mM HEPES (Mediatech) and mechanically homogenized. The resulting cell suspension was washed once with phosphate-buffered saline (PBS, Mediatech). Peripheral blood mononuclear cells (PBMC) were isolated from heparin-anticoagulated blood on Ficoll-Histopaque (Mediatech) density gradients and washed three times with PBS. Paired PBMC and liver biopsy samples were immediately stained for flow cytometry. All other PBMC were cryopreserved in 70% FBS, 20% RPMI1640, and 10% dimethyl sulfoxide (DMSO) (Sigma-Aldrich).

### B cell analysis

HCV-specific memory B cells were identified by flow cytometry using a biotinylated tetrameric complex of the HCV E2 ectodomain (J6 strain)^52^, kindly provided by Dr. Arash Grakoui, Emory University, Atlanta, GA. The tetrameric complexes were prepared by incubating the biotinylated HCV E2 probe with Streptavidin-R-Phycoerythrin (PROzyme, Hayward, CA) at a 4:1 molar ratio.

PBMCs or mononuclear lymphocytes from liver biopsies were stained with the HCV E2 tetramer for 30 min at room temperature, followed by staining with a panel of surface markers consisting of anti-CD19 Brilliant Ultraviolet (BUV805) (clone HIB19, catalog number 742007, BD Biosciences, 1:320 dilution), anti-IgD BUV563 (clone IA6-2, catalog number 741394, BD Biosciences, 1:160 dilution), anti-CD4 BUV496 (clone SK3, catalog number 564651, BD Biosciences, 1:40 dilution), anti-CD20 Allophycocyanin-H7 (APC-H7) (clone 2H7, catalog number 560734, BD Biosciences, 1:100 dilution), anti-C-X-C Motif Chemokine Receptor 5 (CXCR5, catalog number 747111, BD Biosciences, 1:640 dilution) BV750 (clone RF8B2), anti-IgG BV605 (clone G18-145, catalog number 563246, BD Biosciences, 1:20 dilution), anti-CD10 BV510 (clone HI10a, cat563032, BD Biosciences, 1:10 dilution), anti-C-X-C Motif Chemokine Receptor 3 (CXCR3) Phycoerythrin (PE)-CF594 (clone 1C6/CXCR3, catalog number 562451, BD Biosciences, 1:80 dilution) (BD, San Jose, CA), anti-CD21 Fluorescein isothiocyanate (FITC) (clone Bu32, catalog number 354910, BioLegend, 1:100 dilution), anti-CD27 peridinin chlorophyll-A protein cyanine 5.5 (PerCPCy5.5) (clone O323, catalog number 302820, BioLegend, 1:67 dilution), and LIVE/DEAD Fixable Aqua Dead Cell Stain Kit (Thermo Fisher Scientific) for 20 min at 4 °C. All samples were immediately acquired on a BD FACS Symphony flow cytometer using FACS Diva Version 6.1.3 (BD). Data were analyzed using FlowJo version 10.4.2 (Tree Star, Ashland, OR). Samples with less than 30 tetramer-positive events were excluded from further analysis.

For functional analysis, B cells were differentiated into antibody-secreting cells^80^. Briefly, irradiated 3T3-ms-CD40L feeder cells (kindly provided by Dr. Mark Connors, NIAID, Bethesda, MD) were seeded in 96-well U bottom plates in Iscove’s Modified Dulbecco’s Medium (IMDM, Thermo Fisher Scientific) containing 10% ultra-low IgG FBS (Thermo Fisher Scientific). Two days later, B cells were purified to >95% from PBMC using the EasySep Human Pan-B cell Enrichment Kit (Stemcell Technologies, Cambridge, MA), resuspended in IMDM containing insulin (5 μg/ml; Sigma-Aldrich), transferrin (50 μg/ml; Sigma-Aldrich), Interleukin (IL)−2 (200 IU/ml; Sigma-Aldrich) and IL-21 (50 ng/ml R&D Systems, Minneapolis, MN) and added to the 3T3-ms-CD40L feeder cell culture. On days 3, 5, 7, and 9 of the culture, half of the medium was removed and a fresh medium with cytokines was added.

ELISpot plates were treated with 35% ethanol, washed with PBS, and coated with monoclonal antibody to human IgG (clone MT91/145, cat3850-3-250, Mabtech Inc., Cincinnati, OH, 1:50) at 4 °C overnight. After blocking with 10% ultra-low IgG fetal bovine serum in IMDM, B cells from the 11-day cultures were added in fresh IMDM containing insulin (5 μg/ml), transferrin (50 μg/ml), IL-2 (200 IU/ml), IL-21 (50 ng/ml), and IL-6 (50 ng/ml; R&D Systems, Minneapolis, MN). Fifty thousand B cells were seeded per well for the determination of HCV E2-specific spots, and 1575 cells per well for the determination of total IgG spots. After a 20-h incubation at 37 °C, B cells were washed off the plate, and the secreted antibodies were detected using biotin-conjugated HCV E2 (1 μg/ml, kindly provided by Arash Grakoui) or biotin-conjugated anti-human IgG monoclonal antibody (clone MT78/145, cat3850-6-250, Mabtech Inc, 1:1000), followed by addition of alkaline phosphatase-conjugated streptavidin (1:1000 dilution; Mabtech Inc.). The assay was developed with 1-Step NBT/BCIP Substrate Solution (Thermo Fisher Scientific). Spots were counted with an AID EliSpot Reader Systems using AID EliSpot Software Version 7.0 (Autoimmun Diagnostika GmbH, Strassberg, Germany).

### Statistical analyses

For the comparison of pretreatment NAb50 values, a linear mixed model based on the log-transform of the outcome measurement was performed and the Tukey–Kramer procedure was used as a post hoc test. For the model fitting to the NAb50 data or the half-life estimation of statistical inference, the data were examined visually to determine a relationship between NAb50 and time. After this examination, we utilized exponential decay models as previously published^38^. However, these models were found to not fit the data adequately. We then used a simple linear model of an exponential, with the dependent variable normalized for each patient’s baseline value: $\frac{{N{{{{{\mathrm{A}}}}}}}{{{{{{\mathrm{b}}}}}}}_{50}}{{{{{{{\mathrm{baseline}}}}}}}}={\beta }_{0}+{e}^{{\beta }_{1}{{{{{{\mathrm{time}}}}}}}}$. The adequacy of the fit was checked using a visual inspection of the model’s residuals on plots known to identify the fidelity of the model to its underlying requisite statistical assumptions (e.g., the assumption of homoscedasticity). After estimates for β_0_ and β_1_ were determined, the half-life for each set of experimental conditions was calculated by inverting the original model. The standard error for the half-life estimates was calculated via a bootstrap of at least 500 cycles. With the original half-life estimates and the associated standard errors, this allowed to construct 95% confidence intervals for the differences in half-life between designated groups. Analysis was performed using Wolfram Mathematica, v. 11.0 and SAS 9.4 (TS1M6).

Immunological data were assessed with Wilcoxon signed-rank test for paired samples and the Mann–Whitney *U*-test for unpaired samples, respectively, using GraphPad Prism version 8.0.1 and 8.1.2 (GraphPad Software, La Jolla, CA.) Two-sided *p* values <0.05 were considered significant.

### Reporting summary

Further information on research design is available in the Nature Research Reporting Summary linked to this article.

## Supplementary information

Supplementary Information

Reporting Summary

## Data Availability

The raw data for Figs. 1–6 and Supplementary Figs. 1, 2 are provided in the Source Data file. The HCV E2 monomer must be obtained through an MTA with Arash Grakoui, Emory University. Source data are provided with this paper.

## Source data

Source Data

## Code availability

Code written in R for deconvolution analysis is freely available (GitHub repository: https://github.com/BaileyLabHCV/Neutralizing-breadth.git).
