# Supplementary material for: Serum neutralization activity declines but memory B cells persist after cure of chronic hepatitis C
Source: Nat Commun. 2022 Sep 16;13:5446. doi: 10.1038/s41467-022-33035-z (PMC9481596; doi:10.1038/s41467-022-33035-z)
Supplement: Supplementary file 1 — Supplementary Information [file 41467_2022_33035_MOESM1_ESM.pdf]

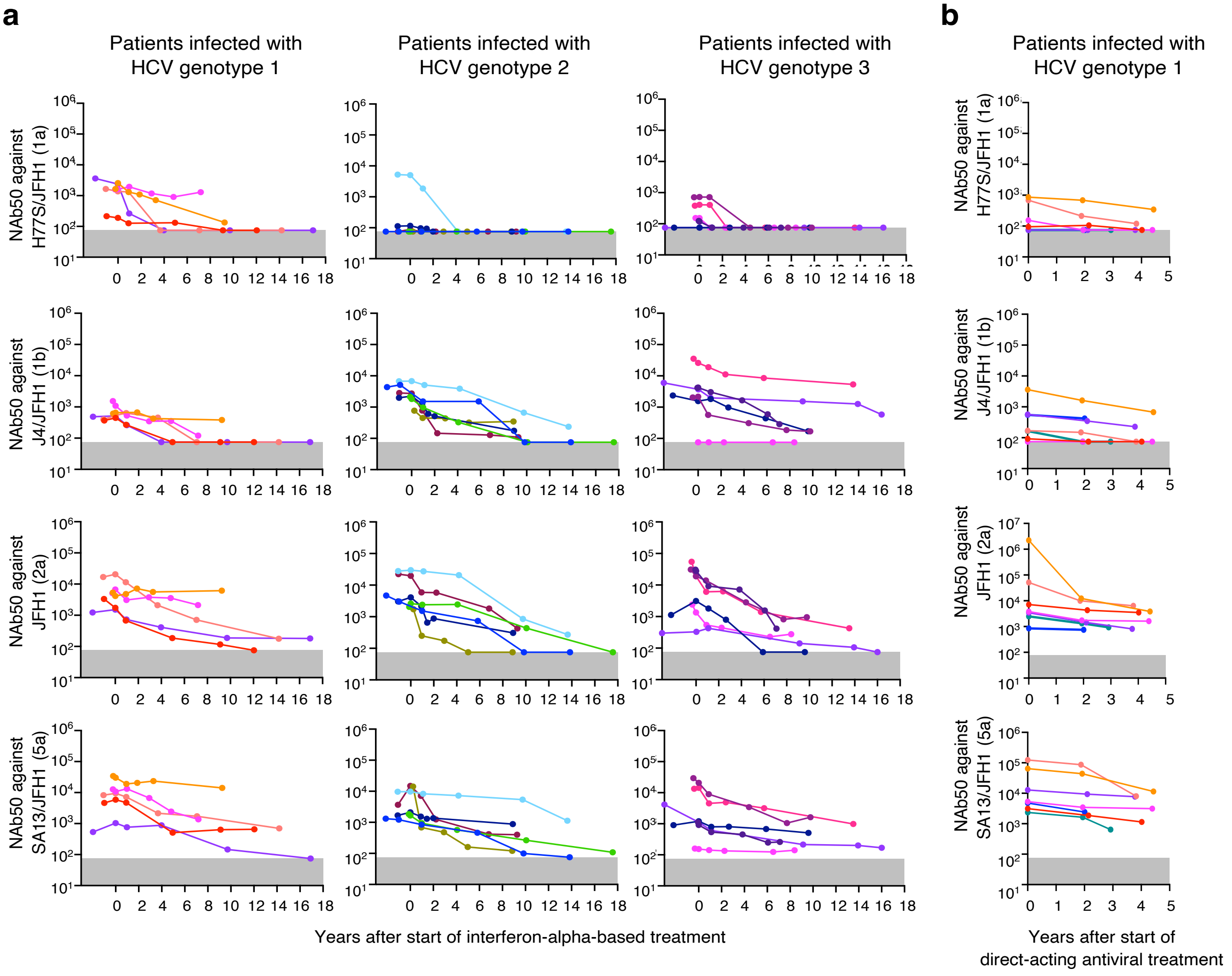

Supplementary Fig 1. HCV-neutralizing activity declines after cure of chronic hepatitis C.

(**a-b**) Serum neutralizing activity was tested against HCVcc with JFH1 backbone and core-NS2 sequences of H77S (genotype 1a), J4 (genotype 1b), JFH1 (genotype 2a), and SA13 (genotype 5a).

a Each data point represents the 50% neutralizing activity (NAb50) from a serum sample that was collected at the indicated time point prior to or after successful interferon-alpha-based therapy of patients with chronic HCV genotype 1 (n =5), HCV genotype 2 (n =6), or HCV genotype 3 infection (n =6).

b Each data point represents the 50% neutralizing activity (NAb50) from a serum sample that was collected at the indicated time point prior to after successful DAA-based therapy of patients with chronic HCV genotype 1 infection (n =7).

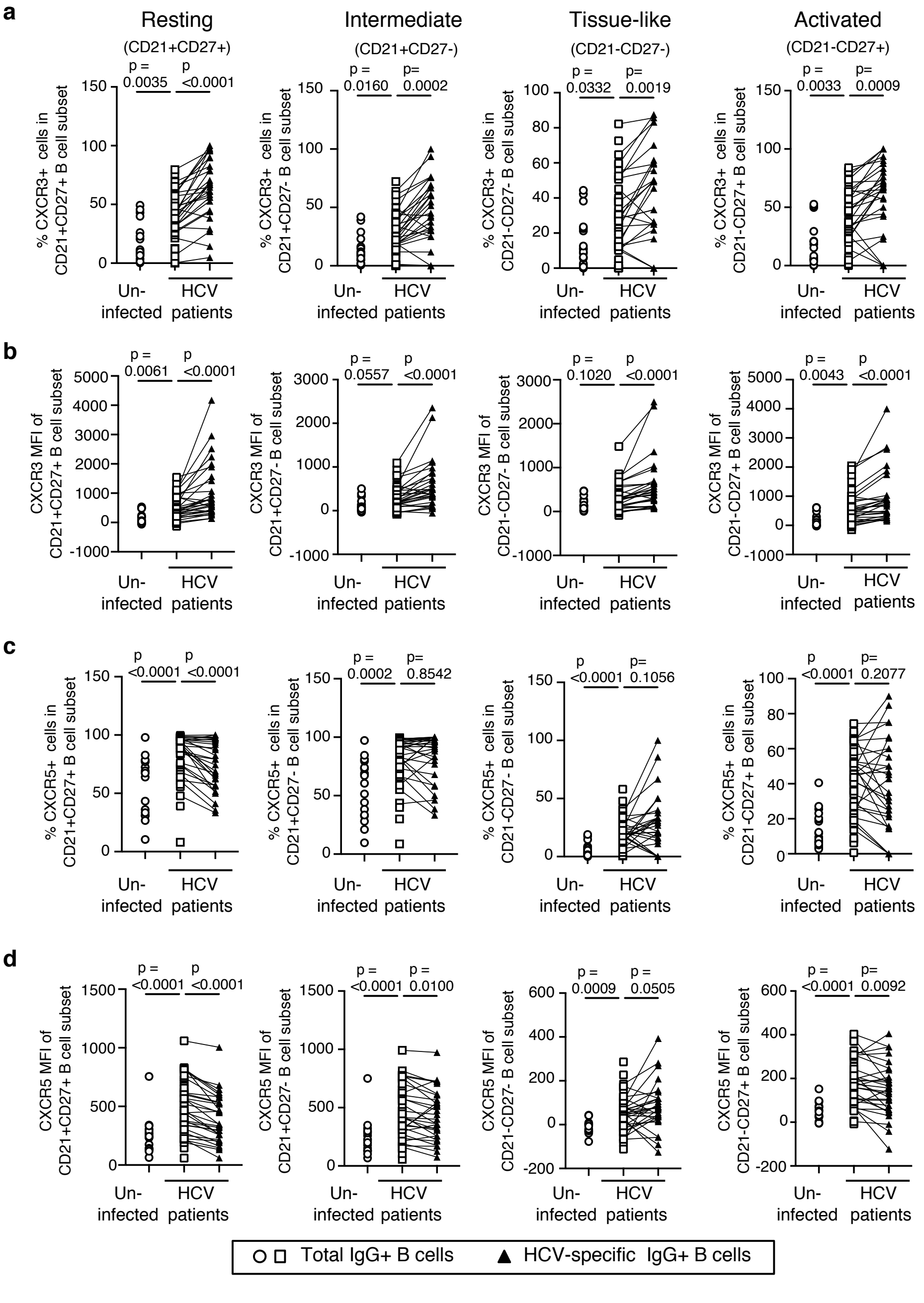

Supplementary Fig 2. Chemokine receptor profile of total and HCV-specific CD21/CD27 memory B cell subsets. (**a, b**) The frequencies of CXCR3+ (liver-homing) (**a**) and the CXCR3 MFI (**b**) of IgG+ B cell subsets from patients with chronic HCV infection (n =44) and uninfected controls (n =17), and of CXCR3+ HCV-specific IgG+ B cells from patients with chronic HCV infection were compared (n =28 patients studied for resting and activated HCV-specific B cells; n =27 patients studied for intermediate HCV-specific B cells; n =26 patients studied for tissue-like HCV-specific B cells). (**c, d**) The frequencies of CXCR5+ (lymph node-homing) (**c**) and the CXCR5 MFI (**d**) of IgG+ B cell subsets from patients with chronic HCV infection (n =44) and uninfected controls (n =17), and of CXCR5+ HCV-specific IgG+ B cells from patients with chronic HCV infection were compared (n =28 patients studied for resting HCV-specific B cells; n =27 patients studied for intermediate and activated HCV-specific B cells; n =26 patients studied for tissue-like HCV-specific B cells). In all panels, B cell subsets were gated based on CD21 and CD27 expression. Two-sided p-values were calculated with the Wilcoxon signed rank test was used for paired data, and the Mann-Whitney U test was used for unpaired data. Each data point represents one study participant. Samples with less than 30 HCV E2 tetramer+ events were excluded.
