# Supplementary material for: Serum neutralization activity declines but memory B cells persist after cure of chronic hepatitis C
Source: Nat Commun. 2022 Sep 16;13:5446. doi: 10.1038/s41467-022-33035-z (PMC9481596; doi:10.1038/s41467-022-33035-z)
Supplement: Supplementary file 2 — Reporting Summary [file 41467_2022_33035_MOESM2_ESM.pdf]

nature research

Corresponding author(s): Barbara Rehermann

Last updated by author(s): 8/18/2022

Reporting Summary

Nature Research wishes to improve the reproducibility of the work that we publish. This form provides structure for consistency and transparency in reporting. For further information on Nature Research policies, see our Editorial Policies and the Editorial Policy Checklist.

Statistics

For all statistical analyses, confirm that the following items are present in the figure legend, table legend, main text, or Methods section.

n/a	Confirmed	
☐	☒	The exact sample size (*n*) for each experimental group/condition, given as a discrete number and unit of measurement
☐	☒	A statement on whether measurements were taken from distinct samples or whether the same sample was measured repeatedly
☐	☒	The statistical test(s) used AND whether they are one- or two-sided<br>*Only common tests should be described solely by name; describe more complex techniques in the Methods section.*
☒	☐	A description of all covariates tested
☐	☒	A description of any assumptions or corrections, such as tests of normality and adjustment for multiple comparisons
☐	☒	A full description of the statistical parameters including central tendency (e.g. means) or other basic estimates (e.g. regression coefficient) AND variation (e.g. standard deviation) or associated estimates of uncertainty (e.g. confidence intervals)
☐	☒	For null hypothesis testing, the test statistic (e.g. *F*, *t*, *r*) with confidence intervals, effect sizes, degrees of freedom and *P* value noted<br>*Give P values as exact values whenever suitable.*
☒	☐	For Bayesian analysis, information on the choice of priors and Markov chain Monte Carlo settings
☒	☐	For hierarchical and complex designs, identification of the appropriate level for tests and full reporting of outcomes
☐	☒	Estimates of effect sizes (e.g. Cohen's *d*, Pearson's *r*), indicating how they were calculated

Our web collection on statistics for biologists contains articles on many of the points above.

Software and code

Policy information about availability of computer code

Data collection	FACS Diva Version 6.1.3 (BD Biosciences), AID EliSpot Software Version 7.0 (Autoimmun Diagnostika GmbH, Strassberg, Germany), BioSpot 5.0 software (Cellular Technology Limited, Shaker Heights, OH)
Data analysis	Prism version 8.0.1 and version 8.1.2 (GraphPad Software, CA), FlowJo version 10.4.2 (TreeStar, Ashland, OR), Wolfram Mathematica, v. 11.0, SAS 9.4 (TS1M6)

For manuscripts utilizing custom algorithms or software that are central to the research but not yet described in published literature, software must be made available to editors and reviewers. We strongly encourage code deposition in a community repository (e.g. GitHub). See the Nature Research guidelines for submitting code & software for further information.

Data

Policy information about availability of data

All manuscripts must include a data availability statement. This statement should provide the following information, where applicable:

- Accession codes, unique identifiers, or web links for publicly available datasets
- A list of figures that have associated raw data
- A description of any restrictions on data availability

The raw data underlying all Figures and Supplemental Figures in this study are provided in the Source Data file.

Field-specific reporting

Please select the one below that is the best fit for your research. If you are not sure, read the appropriate sections before making your selection.

- [x] Life sciences
- [ ] Behavioural & social sciences
- [ ] Ecological, evolutionary & environmental sciences

For a reference copy of the document with all sections, see nature.com/documents/nr-reporting-summary-flat.pdf

Life sciences study design

All studies must disclose on these points even when the disclosure is negative.

Sample size	No statistical analysis was performed to predetermine sample size. The sample size was selected based on similar published studies in the field (Boisvert et al. J Immunol. 2016, Burton et al. J Clin Invest. 2018, Bolte et al. Gastroenterology. 2018) and on available patient samples
Data exclusions	Samples with less than 30 tetramer-positive events were excluded from further analysis of B cells
Replication	Phenotyping of B cells and determination of antibody titers and serum neutralizing activity were performed in independent multiple patients. Experimental variation between donors has been described graphically with all data points displayed in each figure. The techniques used in the experiments (e.g. neutralization assay, flow cytometry, ELISpot, data analysis) were repeatedly performed on independent days. Experiments involving cell culture with Hep3B and Huh7.5.1 cells were all performed in duplicates or triplicates with several independent donors.
Randomization	No randomization was performed as this was not relevant for the study because all immunological analysis in this study was performed for patients who had responded to treatment. B cell response and neutralizing activity were investigated in cryopreserved serial biosamples prior to and after the treatment-induced HCV clearance. HCV-specific memory B cell response and total memory B cell response were compared in paired samples prior to and after HCV clearance.
Blinding	Blinding was not performed because stored sera and PBMCs were selected based on the number of samples available and/or the length of follow up of the patients after treatment-induced clearance of HCV infection. Assays were run independently by multiple researchers and with different assay systems with consistent results.

Reporting for specific materials, systems and methods

We require information from authors about some types of materials, experimental systems and methods used in many studies. Here, indicate whether each material, system or method listed is relevant to your study. If you are not sure if a list item applies to your research, read the appropriate section before selecting a response.

Materials & experimental systems

n/a	Involved in the study	
[ ]	[x]	Antibodies
[ ]	[x]	Eukaryotic cell lines
[x]	[ ]	Palaeontology and archaeology
[x]	[ ]	Animals and other organisms
[ ]	[x]	Human research participants
[ ]	[x]	Clinical data
[x]	[ ]	Dual use research of concern

Methods

n/a	Involved in the study	
[x]	[ ]	ChIP-seq
[ ]	[x]	Flow cytometry
[x]	[ ]	MRI-based neuroimaging

Antibodies

Antibodies used	anti-CD19 (clone HIB19, catalogue number 742007, BD Bioscience, 1:320 dilution),<br>anti-IgD (clone IA6-2 , catalogue number 741394, BD Bioscience, 1:160 dilution),<br>anti-CD4 (clone SK3, catalogue number 564651, BD Bioscience, 1:40 dilution),<br>anti-CD27 (clone O323, catalogue number 302820, Bio Legend, 1:67 dilution),<br>anti-CD21 (clone Bu32, catalogue number 354910, Bio Legend, 1:100 dilution),<br>anti-CD20 (clone 2H7, catalogue number 560734, BD Bioscience, 1:100 dilution),<br>anti-CXCR5 (clone RF8B2, catalogue number 747111, BD Bioscience, 1:640 dilution),<br>anti-IgG (clone G18-145, catalogue number 563246, BD Bioscience, 1:20 dilution),<br>anti-CD10 (clone HI10a, catalogue number 563032, BD Bioscience, 1:10 dilution),<br>anti-CXCR3 (clone 1C6/CXCR3, catalogue number 562451, BD Bioscience, 1:80 dilution),<br>purified IgG (clone MT91/145, catalogue number 3850-3-250, Mabtech, 1:50 dilution),<br>biotinylated IgG (clone MT78/145, catalogue number 3850-6-250, Mabtech, 1:1000 dilution),<br>human serum IgG, catalogue number 4506-10MG, Sigma, 1:20 dilution)

Validation	All antibodies were purchased from well established manufacturers and were validated by the suppliers for species (human), target and application. For antibodies used for flow cytometry, the vendors' websites include flow cytometry blots.

Eukaryotic cell lines

Policy information about cell lines

Cell line source(s)	HEK293T cells and HEP3B cells were kindly provided by Dr. Justin Bailey (John Hopkins University School of Medicine, Baltimore, MD) and originally purchased from ATCC (catalogue numbers CRL-3216 and HB-8084, respectively); The Huh7.5.1 cell line was generated and provided by Dr. Francis V. Chisari, Scripps Research Institute, La Jolla, CA (Zhong et al., Proc Natl Acad Sci U S A. 2005; 102:9294-9)
Authentication	The cell lines were not authenticated.
Mycoplasma contamination	Cell lines tested negative for mycoplasma.
Commonly misidentified lines (See ICLAC register)	No commonly misidentified cell lines were used in the study.

Human research participants

Policy information about studies involving human research participants

Population characteristics	Cryopreserved sera and PBMC who were chronically infected with HCV or had a sustained virological response to treatment of chronic HCV infection were selected based on the number of biosamples available and/or the length of follow up of the patients after treatment-induced clearance of HCV infection. Population characteristics are described in Table 1.
Recruitment	Participants were recruited from the Liver Disease Branch outpatient clinic, community clinics and self-referral to participate in clinical trials evaluating antiviral therapy for chronic hepatitis C including interferon plus ribavirin, peginterferon plus ribavirin, peginterferon, ribavirin, asunaprevir plus daclatasvir, and sofosbuvir plus velpatasvir. Inclusion- exclusion criteria were similar among studies and all studies excluded patients with decompensated liver disease or liver cancer. The one notable difference among studies, which should not bias results was that studies of direct acting antiviral agents (asunaprevir, daclatasvir, sofosbuvir and velpatasvir) permitted treatment-experienced patients whereas peginterferon-based studies excluded previously treated patients.
Ethics oversight	NIDDK/NIAMS institutional review board. Informed consent was obtained from all human research participants.

Note that full information on the approval of the study protocol must also be provided in the manuscript.

Clinical data

Policy information about clinical studies

All manuscripts should comply with the ICMJEguidelines for publication of clinical research and a completedCONSORT checklist must be included with all submissions.

Clinical trial registration	Biosamples were collected under NCT00001729, NCT00028093, NCT02468648, NCT01888900, NCT00001971. and NCT03520660
Study protocol	ClinicalTrials.gov
Data collection	Participants were recruited from the Liver Disease Branch outpatient clinic, community clinics and self-referral. All studies were conducted in the outpatient clinical of the Warren Magnuson Clinical Center. Recruitment occurred between October 1997 to September 2018. Recruitment is still ongoing for NCT03520660, and we are reporting baseline data (before start of antiviral therapy of patients that were recruited to the treatment phase of the trial). Data were collected between October 1997 to May 2021
Outcomes	This manuscript does NOT report results of a randomised clinical trial.

Flow Cytometry

Plots

Confirm that:

- [x] The axis labels state the marker and fluorochrome used (e.g. CD4-FITC).
- [x] The axis scales are clearly visible. Include numbers along axes only for bottom left plot of group (a 'group' is an analysis of identical markers).
- [x] All plots are contour plots with outliers or pseudocolor plots.
- [x] A numerical value for number of cells or percentage (with statistics) is provided.

Methodology

Sample preparation	PBMCs or mononuclear lymphocytes from liver biopsies were stained with the HCV E2 tetramer for 30 min at room temperature, followed by staining with a panel of surface markers consisting of anti-CD19 Brilliant Ultraviolet (BUV805)

(clone HIB19), anti-IgD BUV563 (clone IA6-2), anti-CD4 BUV496 (clone SK3), anti-CD20 Allophycocyanin-H7 (APC-H7) (clone 2H7), anti-C-X-C Motif Chemokine Receptor 5 (CXCR5) BV750 (clone RF8B2), anti-IgG BV605 (clone G18-145), anti-CD10 BV510 (clone HI10a), anti-C-X-C Motif Chemokine Receptor 3 (CXCR3) Phycoerythrin (PE)-CF594 (clone 1C6/CXCR3) (BD, San Jose, CA), anti-CD21 Fluorescein isothiocyanate (FITC) (clone Bu32), anti-CD27 peridinin chlorophyl-A protein cyanine 5.5 (PerCP-Cy5.5) (clone O323) (BioLegend), and LIVE/DEAD Fixable Aqua Dead Cell Stain Kit (Thermo Fisher Scientific) for 20 minutes at 4 °C. All samples were immediately acquired on a BD FACS Symphony flow cytometer using FACS Diva Version 6.1.3 (BD). Data were analyzed using FlowJo version 10.4.2 (Tree Star, Ashland, OR). Samples with less than 30 tetramer-positive events were excluded from further analysis.

Instrument

FACS Symphony (BD Biosciences)

Software

FACS Diva Version 6.1.3

Cell population abundance

Cell sorting was not performed.

Gating strategy

The following gating strategy is used:
1. Gate Singlets (FSC-H [y axis], FSC-A [x axis])
2. Gate Live cells (SSC-A [y axis], Live/Dead channel [x axis])
3. Gate Lymphocytes (SSC-A [y axis], FSC-A [x axis])

Representative dot plots are shown in Fig. 4A and Fig. 5A. The boundary between positive and negative staining population are set based on FMO (fluorence minus one) controls

☒ Tick this box to confirm that a figure exemplifying the gating strategy is provided in the Supplementary Information.
